# Shenlijia Attenuates Doxorubicin-Induced Chronic Heart Failure by Inhibiting Cardiac Fibrosis

**DOI:** 10.1155/2021/6659676

**Published:** 2021-07-16

**Authors:** Xutao Sun, Yunjia Song, Ying Xie, Jieru Han, Fei Chen, Yang Sun, Bowen Sui, Deyou Jiang

**Affiliations:** ^1^School of Basic Medical Sciences, Heilongjiang University of Chinese Medicine, Harbin, China; ^2^Department of Pneumology, First Affiliated Hospital, Heilongjiang University of Chinese Medicine, Harbin, China

## Abstract

Application of the anticancer drug doxorubicin (DOX) is restricted due to its adverse, cardiotoxic side effects, which ultimately result in heart failure. Moreover, there are a limited number of chemical agents for the clinical prevention of DOX-induced cardiotoxicity. Based on the theories of traditional Chinese medicine (TCM) on chronic heart failure (CHF), Shenlijia (SLJ), a new TCM compound, has been developed to fulfill multiple functions, including improving cardiac function and inhibiting cardiac fibrosis. In the present study, the protective effects and molecular mechanisms of SLJ on DOX-induced CHF rats were investigated. The CHF rat model was induced by intraperitoneal injection of DOX for six weeks with the cumulative dose of 15 mg/kg. All rats were then randomly divided into the control, CHF, CHF + SLJ (3.0 g/kg per day), and CHF + captopril (3.8 mg/kg per day) groups and treated for further four weeks. Echocardiography and the assessment of hemodynamic parameters were performed to evaluate heart function. A protein chip was applied to identify proteins with diagnostic values that were differentially expressed following SLJ treatment. The data from these investigations showed that SLJ treatment significantly improved cardiac function by increasing the left ventricular ejection fraction, improving the hemodynamic index, and inhibiting interstitial fibrosis. Protein chip analysis revealed that SLJ upregulated MCP-1, MDC, neuropilin-2, TGF-*β*3, thrombospondin, TIE-2, EG-VEGF/PK1, and TIMP-1/2/3 expressions and downregulated that of MMP-13. In addition, immunohistochemistry and western blot results further confirmed that SLJ promoted TIMP-1/2/3 and inhibited MMP-13 expression. The results of the present study suggest that SLJ was effective against DOX-induced CHF rats and is related to the improvement of heart function and ultrastructure and the inhibition of myocardial fibrosis.

## 1. Introduction

Doxorubicin (DOX) is a broad-spectrum anthracycline anticancer agent that has significant effects on leukemia, lymphoma, and breast cancer [1]. Nevertheless, its clinical application is restricted on account of its dose-dependent and cumulative cardiotoxicity, which results in severe heart failure [2]. Once present, DOX-induced cardiotoxicity is irreversible and eventually develops into chronic heart failure (CHF). CHF is the end-stage manifestation of various heart diseases and is the most common cardiovascular disease. Its morbidity and prevalence are increasing worldwide, which is a relatively serious health problem [3]. Although mitochondrial oxidative stress, cardiomyocyte apoptosis, and the inflammatory response are involved in the development of DOX-induced cardiotoxicity, the precise molecular mechanisms require further clarification [4]. Traditionally, DOX-induced cardiotoxicity has been attributed to cardiomyocyte apoptosis due to the excessive production of activated oxygen in the mitochondria, which exceeds the regulatory function of the heart antioxidant defense system [5]. However, endothelial dysfunction, vascular damage, and myocardial fibrosis represent important damage mechanisms to cardiac function following DOX therapy [6–9]. In this regard, the identification of novel therapeutics to alleviate cardiac fibrosis is urgently required.

Over the last few centuries, traditional Chinese medicine (TCM) has been used to regulate homeostasis in the human body, and Chinese herbal medicines have become ubiquitous for the treatment of specific diseases. Some have cardiovascular effects and can be used in combination with pharmaceutical drugs. Moreover, in the last few decades, TCM compounds have been widely accepted as treatments of cardiovascular diseases in China, as they exert multiple targeted and comprehensive conditioning effects [10, 11]. In TCM, it is believed that the heart Qi is the power that maintains blood circulation and keeps blood in the vascellum. Heart failure is primarily caused by Qi deficiency, followed by blood stasis and drowning. To relieve heart failure, it is necessary to restore the heart Qi, reactivate the blood, and disinhibit water flow [12]. The heart is physiologically associated with and thus can pathologically influence other organs during heart failure; therefore, the treatment principles of TCM are to accelerate blood circulation, disinhibit water flow, and disperse swelling by invigorating the heart Qi and warming the Yang.

The SLJ formula was developed from a well-known capsule of Tingli Dazao, according to the principles of TCM. SLJ contains the following seven commonly used herbs: Ren Shen, Hong Jing Tian, Ting Li Zi, Wu Jia Pi, San Qi, Shan Zhu Yu, and Xian Ling Pi. Among these herbs, based on the Chinese Pharmacopoeia [13], Ginseng is a classical TCM for supplying Qi and warming the Yang, and Rhodiola enhances these effects. Sanchi is often used for promoting blood circulation, removing blood stasis, and relieving pain and swelling. Semen Lepidii has the effect of dispelling phlegm and improving edema. Acanthopanax is traditionally used for dispelling wind, improving kidney function, strengthening the bones, and removing blood stasis. Epimedium nourishes the kidney Yang, reinforces the muscles and bones, and relieves rheumatism. Finally, Macrocarpium fills the blood and replenishes the liver and kidneys. In addition, the bioactivity and pharmacological actions of these herbs have been scientifically studied in the past decades. Ginseng has been reported to improve cardiac function, relieve arrhythmia, and alleviate apoptosis associated with heart failure [14]. Additionally, Ginseng relieves DOX-induced cardiotoxicity by inhibiting apoptosis in mice, demonstrating its potential for the treatment of DOX-induced myocardial toxicity in a clinical setting [15]. Rhodiola is commonly prescribed for protecting cardiomyocytes against hypoxia-induced necrosis and apoptosis [16], and Sanchi is known to promote angiogenesis and prevent oxidative stress and apoptosis in the ischemic myocardium, both *in vitro* and *in vivo* [17, 18]. Semen Lepidii is one of the most commonly used herbs for CHF, which increases pulse pressure and cAMP efflux in a dose-dependent manner [19]. Acanthopanax has shown remarkable anti-hypertensive and anti-inflammatory effects [20], while Macrocarpium possesses antiarrhythmia and antioxidant properties [21]. Additionally, Epimedium is widely used in the clinic to stimulate angiogenesis, prevent endoplasmic reticulum stress, and inhibit left ventricular dysfunction, myocardial remodeling, and apoptosis in CHF rats [22–24].

The multiple pharmacological effects of each herb in the SLJ capsule are thought to be effective for the treatment of CHF and include improving cardiac function, reducing myocardial apoptosis, inhibiting ventricular remodeling, and promoting neovascularization. Therefore, it was speculated that the SLJ formula was likely to be cardioprotective in DOX anti-tumor treatment, which may provide a new means of clinically alleviating DOX-induced cardiotoxicity and CHF. However, there is currently insufficient evidence to support these claims. In the present study, an experimental DOX-induced CHF rat model was constructed to investigate the potential effects and possible underlying mechanisms of SLJ on CHF.

## 2. Materials and Methods

### 2.1. SLJ Preparation

As shown in Table 1, the seven medicinal components of SLJ were purchased from the Pharmacy of the First Affiliated Hospital of Heilongjiang University of Chinese Medicine. The herbs were combined in a TCM decoction container, soaked in 12.5 v/w distilled water for 30 min, boiled for 60 min, and extracted 3 times. The extract was filtrated using a G4 filter and concentrated in a 60°C water bath. The final extract (3 g/mL) was pasteurized, bottled, and stored at 4°C.

### 2.2. HPLC Fingerprint

One gram of SLJ capsule was sonicated in 10 mL of ethyl alcohol. The extract was passed through a 0.45 *μ*m PTFE filter before the HPLC injection. After that, the supernatant was collected and analyzed for chemical fingerprint analysis with Agilent HPLC 1200 system (Agilent, Germany). The samples were achieved on a PLATISIL C18 column (4.6 mm × 250 mm; 5 *μ*m). The detection wavelength was set to 280 nm, and the flow rate was 0.8 mL/min [25].

### 2.3. Animals

Male Wistar rats weighing 180–200 g were purchased from Liaoning Changsheng Biotechnology Co., Ltd. (Animal license number: SCXK (liao) 2015–0001). The animals were raised with standard food and water at 25 ± 1°C and 50 ± 5% humidity, with a 12 h light/dark cycle. All animal testing procedures were conducted in accordance with the Ethics Committee Guidelines on the Care and Use of Laboratory Animals (NIH Publications, no. 85–23, revised 1996). The CHF rats were established by intraperitoneal injection of DOX (2.5 mg/kg body weight) once a week for six weeks [26]. Following the last injection, 6 rats from the control and DOX groups were randomly selected to evaluate cardiac function by echocardiography. Compared with the control group, DOX group ejection fraction (EF) and shortening fraction (FS) were significantly reduced, and left ventricular end-diastolic and end-systolic internal diameters (LVIDd/LVIDs) were significantly higher in the DOX group, indicating that the DOX-induced CHF rat model was successfully constructed [27]. Subsequently, all rats were randomly assigned to the control, model, SLJ (3.0 g/kg per day), or captopril treatment group (3.8 mg/kg per day). The model and control group received equal volumes of 0.9% physiological saline. All drugs (including saline) were administered intragastrically once daily for four weeks.

### 2.4. Echocardiography

Echocardiography and subsequent analysis were performed by the same experienced researcher in a blinded manner. Following anesthetization with intraperitoneal injection of 3% sodium pentobarbital solution (30 mg/kg), the rats were fixed in a low-motion position, and the long axis of the parasternal left ventricular was located with a 7–12 MHz phased-array transducer (Vivid 7; GE Healthcare). Detection indexes included the LVIDd/LVIDs, EF, and FS. The average of ≥3 measurements was calculated for each rat.

### 2.5. Hemodynamics

While the animals were still anesthetized, a tip-transducer catheter (diameter, 1 mm) was inserted into the right common carotid artery to assess the following hemodynamic parameters: left ventricular end-diastolic pressure (LVEDP), left ventricular systolic pressure (LVSP), maximum pressure positive, and negative velocity (±dp/dtmax). The results were then analyzed using PowerLab software (30 series; ADInstruments Shanghai Trading Co.). After hemodynamic measurements were acquired, the rats were then euthanized by anesthetic overdose. The hearts were rapidly removed, divided into two parts at the midpoint of the left ventricular long axis, and reserved for histological analysis.

### 2.6. Myocardium Tissue Processing

After the above experiment, part of the apical myocardium was excised and fixed with formalin for histomorphological assessment. Rat's left ventricular myocardium tissues were lysed with lysis buffer (50 mM Tris base, 1 mM EDTA, 150 mM NaCl, 1% NP-400, 25% sodium deoxycholate, protease inhibitor cocktail, and PH 7.4). Protein concentration was determined using a BCA protein assay kit (Pierce; Thermo Fisher Scientific, Inc.) according to the manufacturer's instructions.

### 2.7. Transmission Electron Microscopy

To analyze the effect of SLJ on the heart tissue ultrastructure, transmission electron microscopy was performed. Fresh myocardial tissue samples from the apex cordis were fixed overnight with 2.5% glutaraldehyde at 4°C and washed 3 times in PBS. The tissues were dehydrated and embedded in epoxy resin and subsequently solidified at 60°C for 2 h. Each heart was cut into 2 ultrathin sections and stained with 0.2% lead citrate and 1% uranyl acetate. Images were obtained at different magnifications using a JEM-1400PLUS transmission electron microscope (JEOL, Ltd.).

### 2.8. Hematoxylin-Eosin (HE) Staining

Myocardial specimens were rinsed with phosphate buffer saline (PBS) and fixed overnight with 4% paraformaldehyde at 4°C. The specimens were then dehydrated with 75%, 95%, and 100% alcohol, treated with xylene, and embedded in paraffin. HE staining was performed using serial tissue sections (4 *μ*m each), and histopathological changes to the myocardial tissues were evaluated with an optical microscope (×400).

### 2.9. Antibody Arrays

The relative expression levels of 90 proteins extracted from the myocardial tissues of the control, model, and SLJ group were detected using cytokine antibody arrays (Rat Antibody L-Series 90 Array) in accordance with the manufacturer's protocol. Briefly, chips containing 90 different antibodies were sealed with blocking buffer and subsequently incubated with the protein samples. The chips were then washed and incubated with biotin-labeled antibodies against these different proteins. Subsequently, the chips were incubated with a horseradish peroxidase-labeled anti-streptomycin biotin antibody, developed with enhanced chemiluminescence reagents (Cell Signaling Technology, Inc.), and exposed to autoradiographic film (BioMax Lite; Kodak). The films were scanned, and the images were converted to pixel densities; relative protein expression was analyzed by subtracting the background intensities and comparing these with the positive control spots.

### 2.10. Immunohistochemistry

Immunohistochemical staining was performed to detect the protein expression levels of collagen I, collagen III, matrix metalloproteinase MMP-13, and metalloproteinase inhibitors TIMP-1, TIMP-2, and TIMP-3. Tissues were embedded as described above. The paraffin-embedded myocardial tissues were cut into 4 µm sections and placed on slides. The sections were deparaffinized, dehydrated, and incubated in 3% H_2_O_2_ at 25°C for 10 min. Following incubation with pepsin at 37°C for 30 min to restore antigen activity, blocking was performed with 5% goat serum (Invitrogen, Co., USA) at room temperature for 30 min. Next, the sections were incubated with primary antibodies rabbit anti-collagen I, MMP-13, TIMP-3 (1:500; Abcam, USA), mouse anti-collagen III (1:1,000; Abcam, USA), TIMP-1 (1:500; Abcam, USA), and TIMP-2 (1:800; Abcam, USA), overnight at 4°C. On the second day, the sections were washed in PBS for 5 min and subsequently incubated with corresponding secondary antibodies (concentration dilution is twice that of the primary antibody) at 37°C for 1 h. The tissue slices were washed with PBS (3 × 5 min) and counterstained with hematoxylin before washing thoroughly. Images were obtained using an optical microscope at ×400 magnification.

### 2.11. Western Blot

Western blot was performed to determine the levels of collagen I, collagen III, TIMP-1/2/3, and MMP-13 in rat myocardium. The myocardium was ground into a homogenate with RIPA lysis buffer (the mass volume ratio 1:10 (mg/*μ*L)), and then the homogenate was centrifuged for 15 min at 4°C to separate the supernatant [28], and the protein concentration was detected by BCA Kit (Beijing Biyuntian Co., China). The same amount of protein was separated by boiling with 10% SDS-PAGE. The protein gels were then removed to the nitrocellulose membrane by electrophoresis (Amersham, USA). The protein bands were incubated with the primary antibodies for mouse anti-collagen III (1:500; Sigma, USA), MMP-13 (1:400; Sigma, USA), GAPDH (1:2,000; Kangcheng, China), rabbit anti-collagen I (1:1,000; Abcam, USA), TIMP-1 (1:500; Abcam, USA), TIMP-2 (1:1,000; Abcam, USA), and TIMP-3 (1:1,000; CST, USA). The bands were then washed and incubated with the corresponding secondary antibodies (1:3,000; CST, USA) for 1 h. Bands were visualized on a FluorChem *M* MultiFluor System (ProteinSimple, USA) using chemiluminescence detection reagents. Optical density analysis was done with AlphaEaseFC (Alpha, USA).

### 2.12. Statistical Analysis

All statistical analyses were performed using SPSS 17.0 (SPSS, Inc.). One-way ANOVA was used following Tukey's test to compare the differences among four groups, and the Mann–Whitney *U* test was used to compare group differences in the protein tissue array results. *P* < 0.05 was considered to indicate a statistically significant difference.

## 3. Results

### 3.1. Fingerprint of SLJ

**Figure 1:**
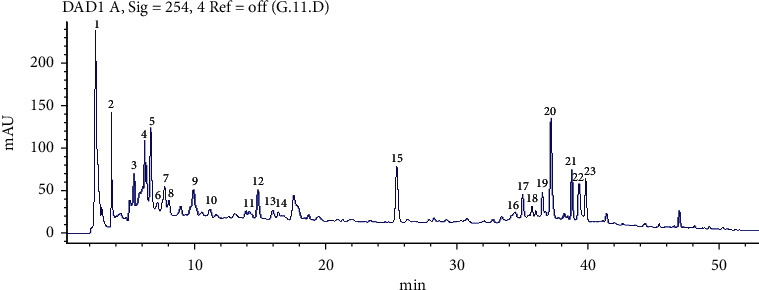
Fingerprint of SLJ. There were mainly 23 eluted peaks being identified.

HPLC chromatograms of 95% ethanol extract of SLJ were obtained, in which there were mainly 23 eluted peaks being identified (Figure 1). The 23 peaks represented major constituents of SLJ extracts with consistent retention values (RSDs of retention times lower than 1% and those of most peak areas lower than 8%). The authenticated 23 peaks, including 37 components of SLJ, were confirmed by comparing retention times with the chemical standards (Table 2).

### 3.2. SLJ Alleviates DOX-Induced Cardiac Dysfunction

**Figure 2:**
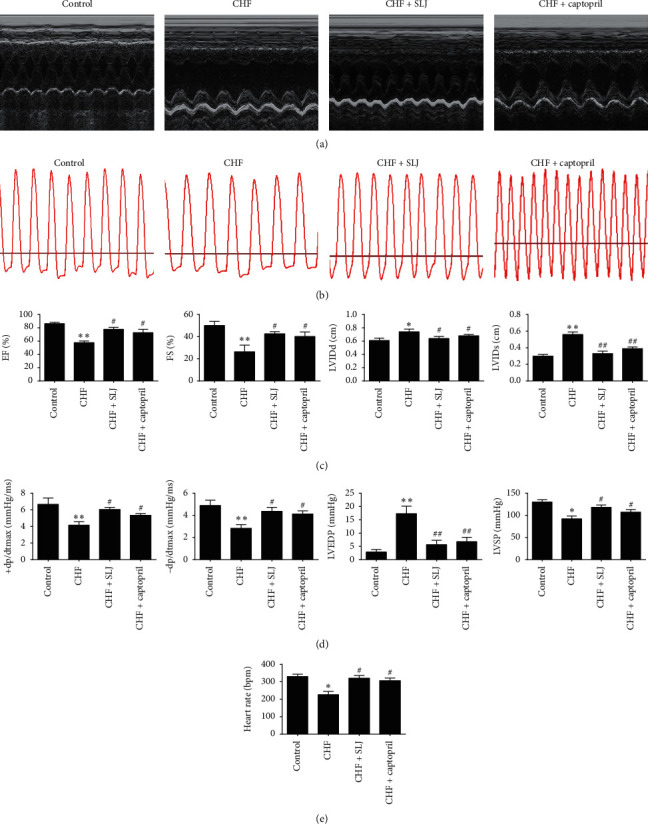
Comparison of echocardiographic and hemodynamic parameters between the control and experimental groups following treatment with SLJ: (a, b) M-mode echocardiographic and hemodynamic images for each group; (c) cardiac systolic functions indicated by EF, FS, LVIDd, and LVIDs were compared among different groups; and (d) comparison of LVSP, LVEDP, +dp/dtmax, and −dp/dtmax hemodynamic values among different groups after treatment with SLJ. Data are expressed as the mean ± SE. *n* = 8. ^*∗*^*P* < 0.05 and ^*∗∗*^*P* < 0.01 vs. the control group. ^#^*P* < 0.05 and ^##^*P* < 0.01 vs. the CHF group. SJL, Shenlijia; EF, ejection fraction; FS, shortening fraction; LVIDd, left ventricular end-diastolic internal diameter; LVIDs, left ventricular end-systolic internal diameter; LVSP, left ventricular systolic pressure; LVEDP, left ventricular end-diastolic pressure; +dp/dtmax, maximum pressure positive velocity; −dp/dtmax, maximum pressure negative velocity; and CHF, chronic heart failure.

Cardiac dysfunction was induced by intraperitoneal administration of DOX (2.5 mg/kg/w) for six weeks, followed by SLJ, captopril, or saline, administered intragastrically for a further four weeks. Echocardiography and the assessment of hemodynamic parameters revealed significant impairment in left ventricular function after DOX infusion, as indicated by increased LVIDd, LVIDs, and LVEDP and decreased EF, FS, LVSP, +dp/dtmax, and −dp/dtmax compared with the control group (Figure 2). Furthermore, SLJ and captopril significantly ameliorated myocardial dysfunction and remodeling, which was verified by increased EF, FS, LVSP, +dp/dtmax, and −dp/dtmax as well as decreased LVIDd, LVIDs, and LVEDP compared with the model group (Figure 2). These results indicate that SLJ treatment improves cardiac function in DOX-induced CHF.

### 3.3. SLJ Preserves the Myocardial Ultrastructure in Rats with DOX-Induced CHF

**Figure 3:**
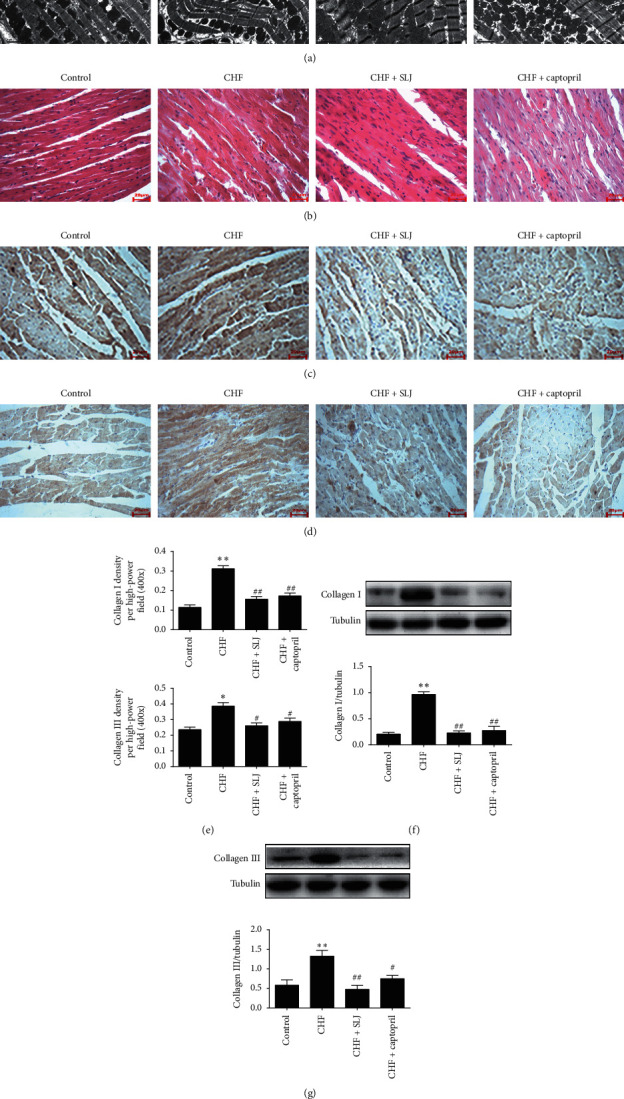
SLJ protects the myocardial ultrastructure and reduces inflammation and fibrosis: (a) representative transmission electron microscopy (×5000) images of the left ventricle; (b) HE staining for inflammatory-cell counts; (c, d) expression of collagen I and III protein in the myocardium using immunohistochemistry; (e, f) western blot analysis of collagen I and III protein expression in myocardium. *n* = 8. ^*∗*^*P* < 0.05 and ^*∗∗*^*P* < 0.01 vs. the control group. ^#^*P* < 0.05 and ^##^*P* < 0.01 vs. the CHF group. SJL, Shenlijia; HE, hematoxylin-eosin; and CHF, chronic heart failure.

Transmission electron microscopy was performed to assess the myocardial ultrastructure of DOX-induced CHF rats (Figure 3(a)). Compared with the control group, the sarcomeres were destroyed, and the mitochondria became swollen and damaged in the CHF group; treatment with SLJ and captopril resulted in an improvement to the myocardial ultrastructure.

### 3.4. SLJ Inhibits Inflammation and Fibrosis in Rats with DOX-Induced CHF

As shown in Figure 3(b), the model group elicited significantly increased inflammatory-cell infiltration in comparison with the control group, which was reduced by SLJ treatment. Collagens I and III, the primary collagen isoforms, were produced by cardiac fibroblasts. Representative immunohistochemical images and western blot showed that collagen I and III expressions in the left ventricle sections were decreased following SLJ and captopril treatment, compared with those of the model group (Figures 3(c)–3(f)). These findings indicate that SLJ suppresses inflammation and fibrosis in rats with DOX-induced CHF.

### 3.5. Antibody Microarray Analysis

**Figure 4:**
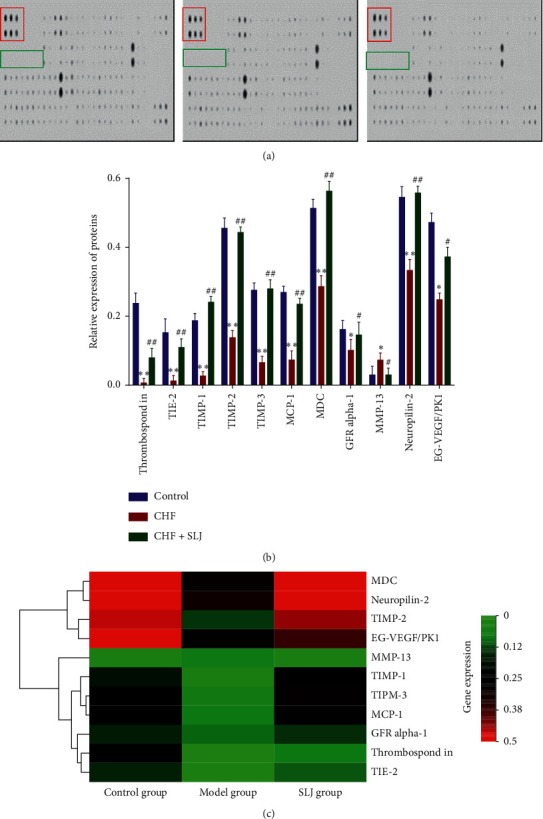
Analysis of antibody microarrays. (a) Image of protein chip data. Red box, positive control; green box, negative control. (b) A total of 11 proteins with significantly different expression levels were associated with SLJ treatment of CHF, as detected by protein array. (c) Heat map of protein microarray data reflecting the expression levels of the 11 proteins. Samples are indicated in columns and proteins in rows. Red, increased expression in SLJ treatment samples compared with CHF samples; green, decreased expression level; black, median expression. *n* = 3. ^*∗*^*P* < 0.05 and ^*∗∗*^*P* < 0.01 vs. the control group. ^#^*P* < 0.05 and ^##^*P* < 0.01 vs. the CHF group. SLJ, Shenlijia; CHF, chronic heart failure.

The expression levels of 90 known proteins (including cytokines, inflammatory factors, chemokines, MMPs, growth factors, and soluble receptors) were measured in the myocardial tissues of all rats. The results showed that 8 proteins were significantly upregulated and 28 were significantly downregulated in the CHF group, compared with the control group. In addition, 14 proteins were significantly upregulated, and 11 were significantly downregulated in the SLJ treatment group, compared with the CHF group (Figure 4(a)). Furthermore, the results of these intergroup comparisons were again compared with each other, revealing that 11 proteins exhibited significantly different expression levels following treatment with CHF with SLJ. Similarities in the abundance of these 11 proteins were sorted by a clustering algorithm, and 3 principal clusters were identified for the control group, and the CHF and SLJ treatment groups, respectively (Figures 4(b) and 4(c)).

### 3.6. SLJ Regulates the Balance between TIMP-1, TIMP-2, TIMP-3, and MMP-13

**Figure 5:**
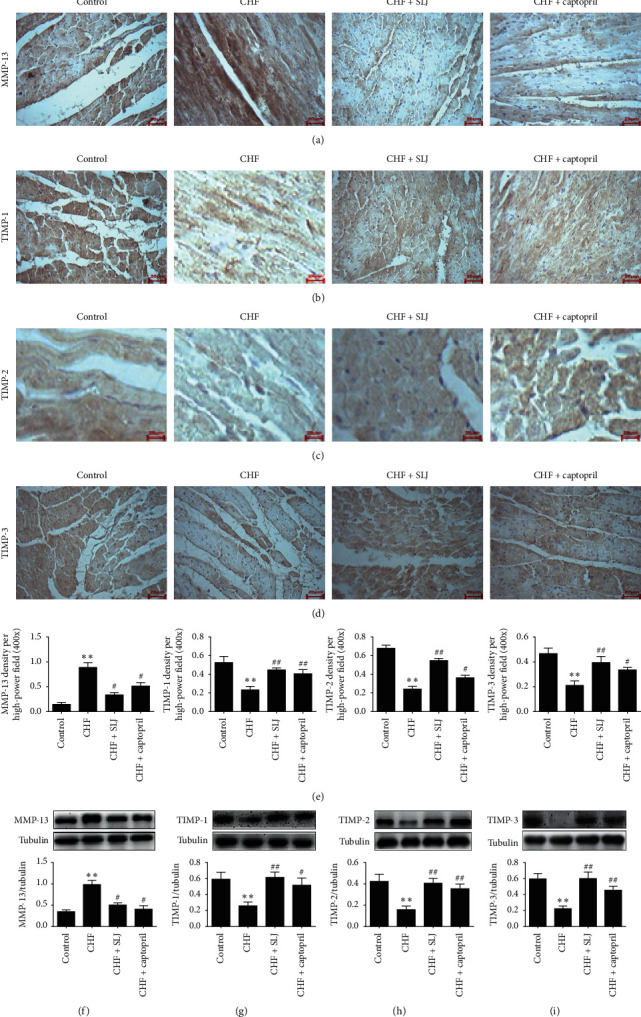
SLJ upregulates the expression of TIMP-1, TIMP-2, and TIMP-3 and downregulates that of MMP-13: (a–d) representative immunohistochemical image analyses of MMP-13, TIMP-1, TIMP-2, and TIMP-3 protein expressions; (e–h) western blot analysis of MMP-13, TIMP-1, TIMP-2, and TIMP-3 protein expressions in myocardium. *n* = 8. ^*∗*^*P* < 0.05 and ^*∗∗*^*P* < 0.01 vs. the control group. ^#^*P* < 0.05 and ^##^*P* < 0.01 vs. the CHF group. SJL, Shenlijia; TIMP, metalloproteinase inhibitor; MMP, matrix metalloproteinase; and CHF, chronic heart failure.

It has been clearly demonstrated that the imbalance between MMPs and their endogenous inhibitors TIMPs plays an important role in left ventricular remodeling. In the present study, analysis of the protein chip results indicated that the expression of MMP-13 was increased in the CHF group, compared with the control group, which was reversed by SLJ and captopril treatment; the expressions of TIMP-1, TIMP-2, and TIMP-3 were the opposite to that of MMP-13. Furthermore, the results of subsequent immunohistochemistry and western blot were consistent with these data (Figure 5). These analyses indicate that SLJ inhibits cardiac remodeling, potentially by regulating the balance of TIMP-1, TIMP-2, TIMP-3, and MMP-13 expression.

## 4. Discussion

DOX is an effective anti-cancer drug with a wide therapeutic range. However, the clinical use of DOX is largely restricted by its side effects, which include cardiotoxicity, cardiac remodeling, and congestive heart failure [3]. Although DOX-induced cardiotoxicity and the pathophysiology of CHF have been widely researched, the underlying molecular mechanisms have not been confirmed, and there are few effective drugs available to prevent DOX-induced cardiopathologies clinically. In this regard, further research remains to be conducted. Nair S et al. found that cathepsin K may be a feasible drug target for the treatment of DOX-induced cardiotoxicity [29]. Based on the TCM theories on CHF therapy [30], the methodology has focused on using native compounds against DOX-induced cardiac toxicity. Previous studies have found that Chinese herbal medicines, date palm pollen extract and liensinine, can alleviate the DOX-induced cardiotoxicity [31, 32]. In the present study, the effects of SLJ, a new herbal compound, against DOX-induced CHF and its potential mechanisms were investigated in rats. The results showed that DOX administration caused heart failure and that SLJ exerted cardioprotection. The primary findings include the followings: (i) SLJ improved cardiac function; (ii) SLJ upregulated MCP-1, MDC, neuropilin-2, TGF-*β*3, thrombospondin, TIE-2, EG-VEGF/PK1, and TIMP-1/2/3 expression and downregulated that of MMP-13; and (iii) SLJ inhibited cardiac fibrosis through the TIMP-1, TIMP-2, and TIMP-3 or MMP-13 signaling pathway.

Some studies have shown that captopril (angiotensin-converting enzyme inhibitor) has a protective effect against DOX-induced cardiotoxicity and is commonly used as a reference drug in the study of Chinese herbal medicine for DOX-induced cardiotoxicity [33]. In the present study, the CHF model was constructed using a 15 mg/kg DOX cumulative dose, and captopril was used as a reference drug to evaluate the protective effect of SLJ on DOX-induced heart failure. The hemodynamic parameters (that included + dp/dtmax, −dp/dtmax, and LVSP) were significantly lower, and LVEDP was markedly higher in the CHF group, compared with the control group; these effects were reversed by SLJ and captopril treatment. Moreover, echocardiographical results showed that SLJ and captopril treatment significantly increased EF and FS and markedly decreased LVIDd and LVIDs. These data provided a basis for the SLJ treatment of DOX-induced CHF.

Extracellular matrix (ECM) remodeling plays an important role in myocardial injury and repair, and regulatory ECM proteins are associated with the progression of various angiocardiopathies. Increased ECM deposition is a mark of cardiac hypertrophy, ischemic cardiomyopathy, and heart failure [34–37]. MMPs and TIMPs are the main regulators of the ECM. The causal relationship between the mediation and stimulation of MMPs and unfavorable alterations in cardiac structure and function has been clearly studied [38, 39]. Thus, regulating the induction and activation of MMPs and their inhibitors (TIMPs) may be an underlying target for the treatment of angiocardiopathy.

TIMPs consist of 4 unique molecules (TIMP-1-4) that differ in the time of expression after tissue damage and that may have a significant impact on the activity of MMPs and influence the proliferation and survival of fibroblasts. The first discovered TIMP, TIMP-1, was described in the late 1970s, and TIMP-4 was initially identified in the late 1990s; these proteins were initially considered to possess comparable structures that could bind to the active form of MMPs at a 1:1 stoichiometry ratio [40, 41]. TIMPs may be involved in multiple cellular processes including propagation and apoptosis and inhibit MMP functioning [42]. MMPs comprise a diverse family of enzymes, including collagenases, stromelysins, gelatinases, and membrane prototype MMPs, all of which possess unique functionality. Multiple animal and clinical studies have uniformly demonstrated alterations and divergences in MMP and TIMP expressions and induction in myocardial remodeling [43, 44]. In postmyocardial infarction remodeling, strong stimulation of MMPs was observed, specifically those linked with inflammation, though this was not necessarily accompanied by a corresponding increase in TIMPs. Specifically, the expressions of TIMP-1 and TIMP-2 increased at the early stages and were subsequently reduced following myocardial injury, while the relative TIMP-4 concentrations declined in the period directly after postmyocardial injury. By contrast, multiple MMPs are both robustly and persistently expressed [45]. Moreover, the increased inconsistency between MMP and TIMP levels is further instantiated when taking the stoichiometric ratio of MMP/TIMP into consideration, and the relative disorder between MMP and TIMP levels indicates the imbalance of this ECM proteolytic system following myocardial injury [46].

In the present study, TIMP-1, TIMP-2, and TIMP-3 expressions were found to be decreased, and MMP-13 expression was increased in the CHF group, compared with the control group, which was subsequently reversed by SLJ treatment. Moreover, compared with the control group, the expression levels of collagens I and III were increased in the CHF group, which was reduced by SLJ administration. These results suggest that SLJ protects the heart from DOX-associated toxicity by inhibiting myocardial fibrosis and remodeling at least partially via the TIMP-1/2/3 or MMP-13 signaling pathway.

To the best of our knowledge, the present study revealed for the first time that SLJ, a new TCM compound with multiple curative effects, significantly improves cardiac function and inhibits cardiac fibrosis, as evidenced by an upregulation in +dp/dtmax, −dp/dtmax, LVSP, EF, and FS and a downregulation in LVEDP, LVIDd, and LVIDs, regulating the balance of TIMP-1, TIMP-2, TIMP-3, and MMP-13. These results indicate that SLJ may be a potential new treatment for the prevention of DOX-induced cardiac damage. However, further research is required to confirm the potential molecular mechanisms involved.

## Figures and Tables

**Table 1:** Components of SLJ.

Number	Identification	Formula
1	Heterodendrin	C_11_H_19_O_6_
	Isoquercitrin	C_21_H_20_O_12_
2	Ginsenoside Rb1	C_54_H_92_O_23_
	Rosmarinic acid	C_18_H_16_O_8_
3	p-Cymene	C_10_H_16_
	Ginsenoside Rg1 + O	C_42_H_72_O_15_
4	20(R)-Ginsenoside F1	C_36_H_62_O_9_
	Descurainolide B	C_21_H_22_O_8_
5	*γ*-Terpinene	C_10_H_16_
6	7*α*-O-Methyl morroniside	C_18_H_28_O_11_
	20(R)-Ginsenoside Rh1	C_36_H_62_O_9_
7	Gallic acid	C_7_H_6_O_5_
	Ginsenoside Rg1 + 2glc	C_54_H_92_O_24_
8	Ginsenoside Rg6	C_42_H_70_O_12_
9	Magnoflorine	C_20_H_24_O_4_
	Ginsenoside Rg1-glc	C_36_H_62_O_9_
	d-Limonene	C_10_H_16_
10	Ginsenoside Rg1-glc isomer	C_36_H_62_O
	7*α*-O-Ethylmorroniside	C_19_H_30_O_11_
11	Ginsenoside Rg1-H_2_O isomer	C_36_H_60_O_8_
12	Protopanaxatriol	C_30_H_52_O_4_
	4-Terpineol	C_10_H_18_O
13	Ginsenoside Rb1-3glc	C_36_H_62_O_8_
14	p-Hydroxyphenacyl-*β*-D-glucopyranoside	C_14_H_18_O_8_
	Ginsenoside Rb1-4glc + O	C_30_H_52_O_4_
15	trans-Nerolidol	C_15_H_26_O
16	Thunbergene	C_20_H_32_
17	n-Hexadecanoic acid	C_16_H_32_O_2_
	Sagittatoside A	C_33_H_40_O_15_
18	Sagittatoside B	C_32_H_38_O_14_
19	2-Phenylethyl-1-*β*-D-glucoside	C_14_H_20_O_6_
	Epimedoside	C_37_H_44_O_17_
20	Epimedigrandioside A	C_39_H_46_O_18_
	12-Octadecadienoic acid	C_18_H_32_O_2_
21	Ethyl gallate	C_9_H_10_O_5_
22	Anhydroicaritin	C_21_H_20_O_6_
23	Desmethylanhydroicaritin	C_20_H_18_O_6_

**Table 2:** Composition of Shenlijia decoction.

Chinese name	Latin name	English name	Quantity (g)
Ren Shen	Panax ginseng	Ginseng	30
Hong Jing Tian	Rhodiola rosea	Rhodiolae crenulatae	15
Ting Li Zi	Semen lepidii	Seed of pepperweed	20
Wu Jia Pi	Acanthopanax senticosus	Slenderstyle acanthopanax bark	20
San Qi	Panax notoginseng	Notoginseng radix et rhizoma	15
Shan Zhu Yu	Fructus corni	Common macrocarpium fruit	15
Xian Ling Pi	Epimedii folium	Epimedium	15

## Data Availability

The data sets used and/or analyzed during the current study are available from the corresponding author on reasonable request.
